# The expression of autophagy-related gene CXCL12 in endometriosis associated ovarian cancer and pan-cancer analysis

**DOI:** 10.3389/fendo.2025.1450892

**Published:** 2025-03-17

**Authors:** Mingwei Yuan, Sijing Chen, Zelan Liao, Kana Wang

**Affiliations:** ^1^ Department of Obstetrics and Gynecology, West China Second University Hospital, Sichuan University, Chengdu, China; ^2^ Key Laboratory of Birth Defects and Related Diseases of Women and Children (Sichuan University), Ministry of Education, Chengdu, China

**Keywords:** endometriosis-associated ovarian cancer, autophagy, integrated bioinformatics approaches, pan-cancer, survival analysis, immune infiltration

## Abstract

**Background:**

Endometriosis-associated ovarian cancer (EAOC), an aggressive form of malignant ovarian neoplasm with origins in endometriosis (EM), has risen to prominence recently. Despite extensive investigation, the precise pathophysiology remains elusive.This article explores new autophagy-related DEG genes between EM and EAOC, and investigates CXCL12’s expression and prognostic relevance across pan-cancer.

**Methods:**

From Gene Expression Omnibus (GEO), we retrieved gene sequencing data to uncover DEGs. We carried out enrichment analysis, PPI network construction and explored CXCL12’s multi-database expression and prognostic significance employing the analytical tools of ONCOMINE, PrognoScan, GEPIA, and Kaplan-Meier Plotter. Subsequently, assessing the relationship between CXCL12 expression and immune presence in cancer utilizing GEPIA and TIMER. Lastly, CXCL12, IL17, STAT3, and FOXP3 protein expressions were determined through immunohistochemistry analysis in EAOC, EM, and normal endometrial tissues.

**Results:**

Two DEGs were discovered and enrichment analysis indicated virus-cytokine/receptor interactions, chemokine signaling, and cytokine-cytokine receptor interplay as pivotal in EAOC. Notably, cancerous tissues exhibited reduced CXCL12 levels compared with non-malignant tissues across cancers. CXCL12, IL17, STAT3, Th17/Treg ratio, and FOXP3 expressions were also lower in EAOC than EM and normal tissues. Additionally, CXCL12 expression was related to stage, survival, immune subtype, and molecular classification across cancers.

**Conclusions:**

In conclusion, our study implicates CXCL12 and altered Th17/Treg balance in progression from EM to EAOC. CXCL12 emerges as a predictive marker for cancer progression across various tumors and is associated with inflammatory response.

## Introduction

Endometriosis (EM), an imposing gynecologic disorder, affects approximately 5%-20% of women in childbearing years (1–3). Among those experiencing pelvic pain and infertility, EM prevalence rises to a shocking 30%-50% (4, 5). Its manifestations include tumors, pain, and infertility, severely impacting quality of life and fertility. Like malignant tumors, EM displays traits of invasiveness, adhesivity, and metastasis, heightening concerns about its cancerous potential. In 1925, Sampson first linked EM with ovarian cancer, establishing the diagnostic criteria for Endometriosis Associated Ovarian Cancer (EAOC). These include coexistence of EM and cancer tissue within a lesion, histological correlation between both, and exclusion of other primary tumors. In 1953, ScoR refined these guidelines further, adding histological evidence of EM transition towards malignancy (6). Numerous studies establish stronger links among EM and ovarian cancer, increasing risk of ovarian cancer up to 1.265-2.14 times for EM patients (7, 8), specifically for ovarian clear cell carcinoma (OCCC) and ovarian endometrioid adenocarcinoma (OEC) (9). This risk persists even postmenopausal and EM symptom resolution (10), notably, CKD(chronic kidney disease)history exceeding ten years or primary infertility augments this risk, respectively 2.51 times and 2.72 times higher than non-EM secondary infertility patients (11, 12).

The mechanism of EM evolving into EAOC remains unclear, potentially linked to microenvironmental factors like immune and inflammation responses, excess estrogen, oxidative stress, or specific gene mutations and alterations such as ARID1A, PTEN, PIK3CA and KRAS) (13–17). Autophagy, a highly conserved metabolic process in eukaryotes, plays pivotal roles in stress response and maintaining cellular homeostasis (18). It not only influences the progression of endometriosis (19) but potentially contributes to ovarian cancer through cell behavior changes like drug resistance, dormancy, and stemness maintenance (20).

Consequently, exploring autophagy’s effects on ovarian cancer related to endometriosis is crucial for understanding this correlation and developing novel therapeutic strategies.

## Materials and methods

### Data sources

Utilizing the Gene Expression Omnibus (GEO) genomic database (http://www.ncbi.nlm.nih.gov/geo), keyword searches for “endometriosis” and “endometriosis-associated ovarian cancer” identified two gene expression datasets meeting our selection criteria: GSE57545 and GSE157153. These datasets were sourced fromGPL18671 and GPL17303 platforms respectively (21, 22).

### Selection of differentially expressed genes

Networkanalyst (https://www.networkanalyst.ca/NetworkAnalyst/faces/home.xhtml) was utilized for differential expression gene (DEG) assessment in comparing nonmalignant endometriosis and endometrioma-associated ovarian carcinoma tissue (23). Genes exhibiting an adjusted P-value <0.05 and a log fold change (|logFC|) >1 were deemed DEGs. The Autophagy Database HAMdb2 (http://hamdb.scbdd.com/home/index/) was employed (24), cross-referencing with datasets GSE57545 and GSE157153 to identify autophagy-linked DEGs. The ggplot2 (version 3.3.6) package within R software was utilized to plot the DEG volcano plot. Online tool Funrich (http://funrich.org/) generated the DEG Venn diagram (25).

### Construction of protein-protein interaction networks and GO/KEGG analysis

GeneMANIA (http://www.genemania.org), a database for elucidating protein-protein interaction (PPI) networks, was employed to construct networks encompassing functionally correlated genes from existing genomics and proteomics datasets (26).Gene functional analysis by GO, KEGG enrichment scrutiny was conducted on genes closely connected to CXC chemokine ligand-12 (CXCL12), sourced from STRING utilizing the “clusterProfiler” and “org.Hs.eg.db” packages in R (27).GO and KEGG pathway enrichments were performed with a p-value threshold of < 0.01, showing outcomes in a ggplot2 bubble chart format.

### Differential expression analysis of CXCL12

Data comprising 11,315 cancerous tissue and matched healthy counterparts from TCGA (https://www.cancer.gov/aboutnci/organization/ccg/research/structural-genomics/tcga) and mRNA patterns in 31 GTEx (https://commonfund.nih.gov/GTEx) derived tissues were utilized. We assessed CXCL12 expression levels across these 31 normal and 33 cancer tissues and compared them against their matched controls. Significance testing using log2 transformation and t-tests revealed expression variances significant at a p-value lower than 0.05. Data processing tasks were executed via R software (version 4.2.1, https://www.Rproject.org), incorporating the ‘‘ggplot2’’ package for graphical representation.

### Immunohistochemistry staining of CXCL12

CXCL12 protein quantification was accessed through the CPTAC database via UALCAN (http://ualcan.path.uab.edu/) (28).The Human Protein Atlas (https://www.proteinatlas.org/), a comprehensive database, catalogs protein distributions in human tissues and cells (29).Evaluating CXCL12 protein expression, we accessed immunohistochemistry data obtained from HPA’s repository of 15 tumor and matched normal tissue specimens.

### Analysis of the diagnosis value of CXCL12

ROC analysis on CXCL12 in 33 cancers evaluated its applicability as a diagnostic tool. mRNA expressions of CXCL12 in TCGA and GTEx somatic tumors and their corresponding adjacent healthy tissue were utilized for these assessments. ROC curves were developed using R’s “pROC” package (v1.17.0.1). Additionally, area under curve (AUC), cutoffs, sensitivities, specificities, positive predictive values, negative predictive values, and Youden’s index (YI) were calculated. An elevated AUC reflects superior diagnostic capability. An AUC between 0.5 and 0.7 denotes poor accuracy, 0.7 to 0.9 marks fair accuracy, with an AUC of 0.9 or above signifying stellar accuracy. High YIs indicate more effective patient-screening methods (30, 31).

### Analysis of the relationships between CXCL12 and prognosis

Accessing TCGA downloads enabled us to explore correlations between CXCL12 expression and patient outcomes: overall survival (OS), disease-specific survival (DSS), and progression-free interval (PFI). For survival analysis per cancer type, the Kaplan–Meier method and log-rank test were utilized. We leveraged R packages “survival” and “survminer” for visualization of survival curves. Additionally, “forestplot” was used to determine the pan-cancer association between CXCL12 expression and survival.

### CXCL12 expression in different molecular and immune subtypes of cancers

Investigating associations between CXCL12 expression and subtype classifications within 33 cancers entailed utilization of the “subtype” module of TISIDB, a comprehensive resource that integrates diverse datasets related to tumor-immune system interplay (32). Investigated CXCL12 mRNA expression among unique immunological subtypes: C1 (wound healing), C2 (IFN-g dominant), C3(inflammatory), C4 (lymphocyte deplete), C5 (immunologically quiet), and C6 (TGF-b dominant).

### Analysis of the promoter methylation of CXCL12

UALCAN (http://ualcan.path.uab.edu/), a comprehensive web interface, facilitated in-depth transcription factor (TF) deregulation evaluation of TCGA gene expression profiles. This research application utilized UALCAN to scrutinize promoter methylation status of the chemokine CXCL12 across diverse cancer types.

### The gene set cancer analysis

The GSCALite platform (http://bioinfo.life.hust.edu.cn/web/GSCALite/) synthesizes genomic data on 33 cancers from TCGA with drug response data from GDSC, CTRP, along with GTEx’s normal tissue info, for streamlined gene set evaluation (33).Utilizing this platform, we examined the alterations to well-known cancer-involved pathways in the 33 cancer variations of CXCL12, specifically focusing on TSC/mTOR, RTK, RAS/MAPK, PI3K/AKT, ER, AR, EMT, DDR, Cell Cycle, and Apoptosis pathways.

### Relationship between CXCL12 expression and immunity

CIBERSORT, an agnostic metagenomic tool, derived relative expression scores for 24 immunity cells in 33 tumor samples to define their immunocyte phenotype. Moreover, R “ggplot2” and “ggpubr” packages determined correlation coefficients between CXCL12 and infiltrating immune subset levels. Additionally, co-expression profiles of CXCL12 with immunity-associated genes coding for MHC, immune activation, suppression, chemokines, and receptors, were evaluated.

### Correlation of CXCL12 expression with DNA methylation

UALCAN (http://ualcan.path.uab.edu/) serves as an interactive web portal enabling extensive analysis of TCGA gene expression data. Utilizing UALCAN, this investigation examined the promoter methylation status of CXCL12 across diverse cancer types. cBioPortal (http://www.cbioportal.org/), a comprehensive resource that incorporates TCGA’s tumor gene datasets, provides researchers with multi-dimensional data visualization (34). Utilizing cBioPortal, we mined data from 32 cancers comprising 10,953 samples for further investigation. On “OncoPrint” and “Cancer Type Summaries”, we explored mutations in the CXCL12 gene across various tumor types and their profiling. “OncoPrint” displayed the target gene’s mutation, copy number, and expression patterns via heat maps, while “Cancer Type Summaries” illustrated the gene’s mutation rates in general carcinomas through bar charts.

### Immunogenomic analyses of CXCL12 in the 33 cancers

The GSVA module coupled with ssGSEA analytics investigated the link between CXCL12 expression and tumor infiltrating lymphocytes, immunomodulators, suppressors, MHC molecules, chemokines, and their respective receptors across 33 cancers. This analysis utilized Spearman’s rank correlation method; p-values below 0.05 were designated as statistically significant. Subsequently, ggplot2 package generated heatmap visualizations.

### Immunohistochemistry of 54 clinical cases

We procured FFPE archives from 26 EAOC tissues, 10 EM, and 18 control endometrial tissues at the West China Secondary Hospital. The procedure involved deparaffination, dehydration, citrate buffer antigen retrieval via microwave, 3% H2O2 treatment, and overnight incubation with anti-human CXCL12, IL17, STAT3, FOXP3 antibodies (Proteintech, Wuhan, China). Next, the sections were incubated with secondary antibody at room temperature for 2 hours. DAB kit (Solarbio, Beijing, China, DA-1010) was employed for color development. Randomly selected fields were observed under microscope, scoring the intensity of positive cells on a scale of 0-3 (0 = faint; 3 = strong), and the staining extent as follows: 0 = < 5%; 1 = 5-25%; 2 = 26-50%; 3 = 51-75%; 4 = 76-100%. The final IHC staining score was obtained by multiplying these two values, defining positivity as a score > 2.

## Results

### Identification of differentially expressed genes associated with autophagy DEG

**Figure 1:**
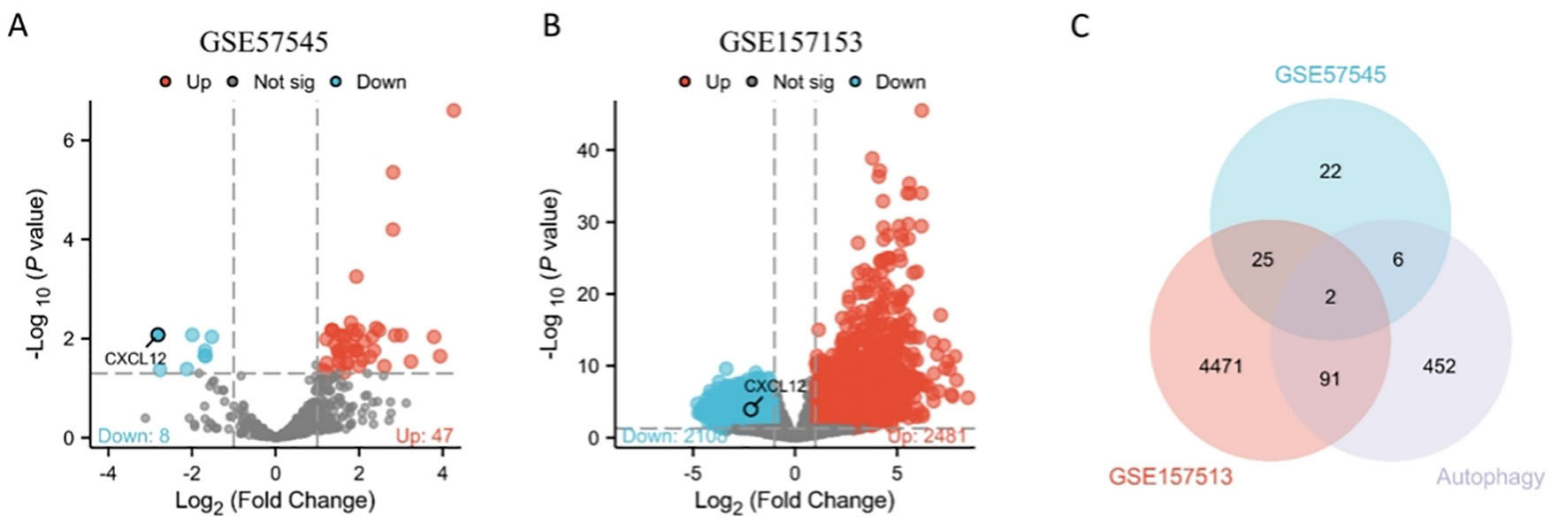
Volcano plots exhibit DEGs of **(A)** GSE57545, **(B)** GSE157153; **(C)** The Venn diagram depicts the common DEGs among GSE57545, GSE157153 and autophagy-related genes.

GSE57545 dataset obtained 130 differentially expressed genes, including 117 up-regulated genes and 13 down-regulated genes, and GSE157153 dataset obtained 3824 differentially genes including 1350 up-regulated genes and 2474 down-regulated genes. Two gene products were identified after intersecting the DEGs in GSE57545, GSE157153, and autophagy-associated genes, and we selected CXCL12 as the differentially expressed genes of autophagy-related genes and further analyzed it as key gene (**Figure 1**).

### Expression landscape of CXCL12

**Figure 2:**
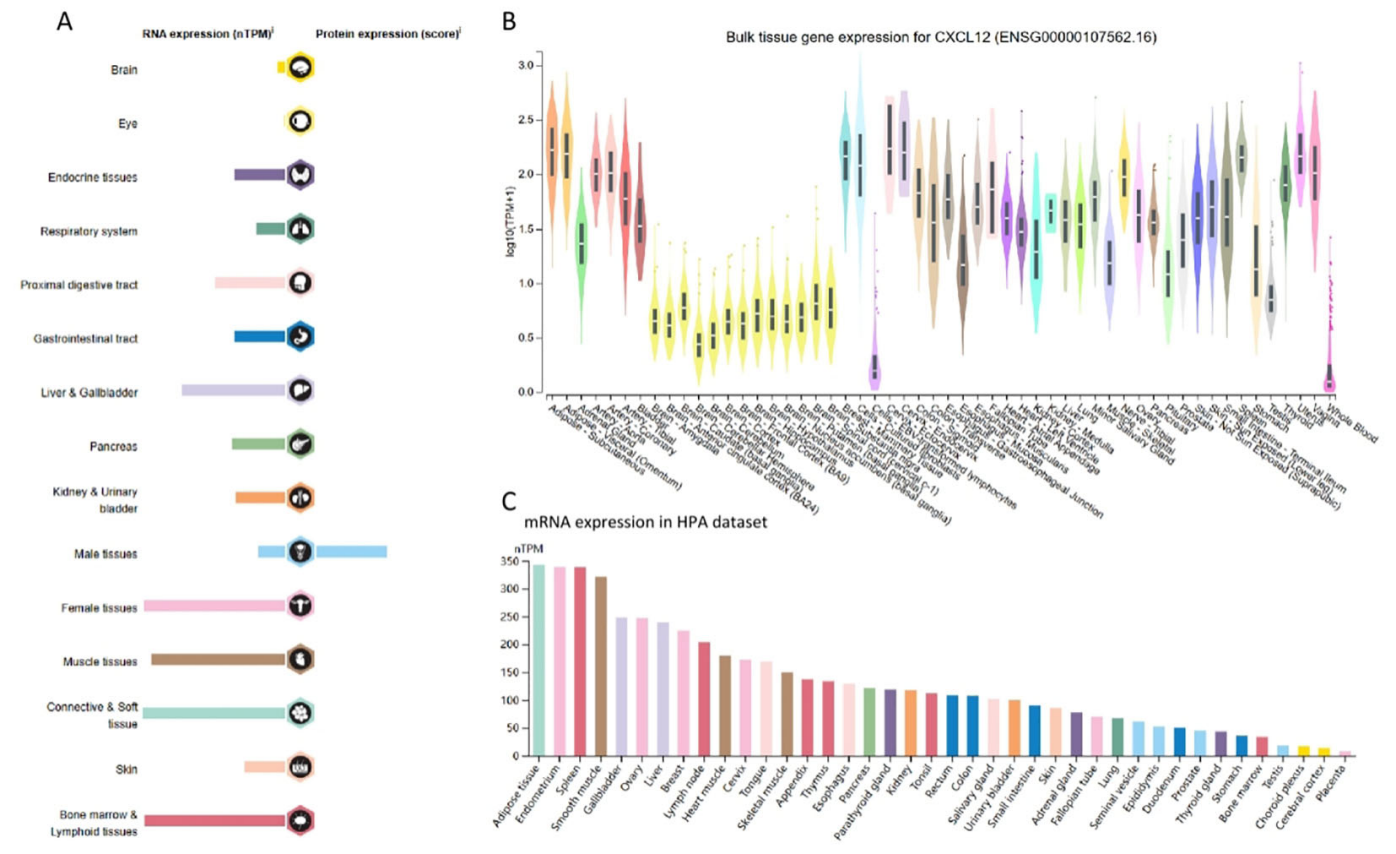
RNA and protein expression profile of CXCL12 in human organs and tissues. **(A)** The summary of CXCL12 mRNA and protein expression in human organs and tissues; **(B)** CXCL12 mRNA expression summary in different human organs and tissues based on GTEx dataset; **(C)** CXCL12 mRNA expression summary in different human organs and tissues based on HPA dataset.

Remarkably, CXCL12 exhibited elevated expression in adipose, endometrial, spleen, and smooth muscle tissues, whereas placental and cerebral cortex showed reduced levels (**Figure 2**).

### Pan-cancer mRNA expression of CXCL12

**Figure 3:**
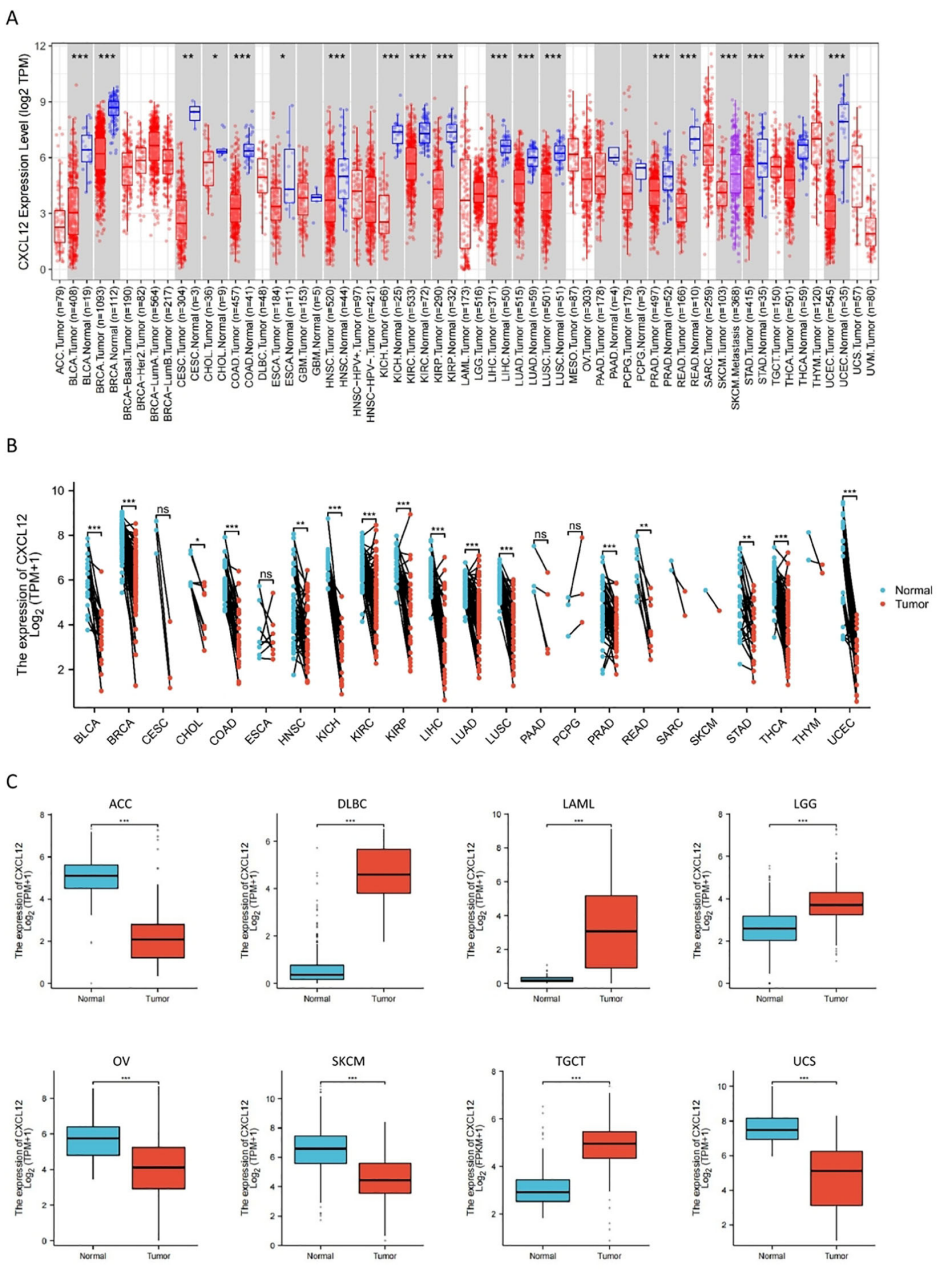
The expression of CXCL12 mRNA in pan-cancer. **(A)** Expression of CXCL12 between the 33 cancers and normal tissues in unpaired sample analysis; **(B)** Expression of CXCL12 between the 33 cancers and normal tissues in paired sample analysis; **(C)** Paired sample analysis of CXCL12 mRNA expression between 8 cancers and normal tissues in ACC, DLBC, LAML, LGG, OV, SKCM, TGCT and UCS. (*p < 0:05, **p < 0:01, ***p < 0:001, ns, Not Significant).

Results indicated reduced CXCL12 levels in tumor compared to their respective control tissues, including BLCA, BRCA, CESC, CHOL, COAD, ESCA, HNSC, KICH, KILC, KILP, LIHC, LUAD, LUSC, PRAD, READ, STAD, THCA, UCEC, ACC, OV, SKCM, UCS. It was increased in DLBC, LAML, LGG, TGCT. When comparing GBM, PAAD, PCPG and their normal tissues, there were no significant differences in CXCL12 gene expression levels. Among the paired sample analyses, CXCL12 mRNA expression was decreased in BLCA, BRCA, CHOL, COAD, HNSC, KICH, KIRC, KIRP LIHC, LUAD, LUSC, PRAD, READ, STAD, THCA, UCEC. There was no difference shown in CESC, ESCA, PAAD, PCPG (**Figure 3**).

### Pan-cancer protein expression of CXCL12

**Figure 4:**
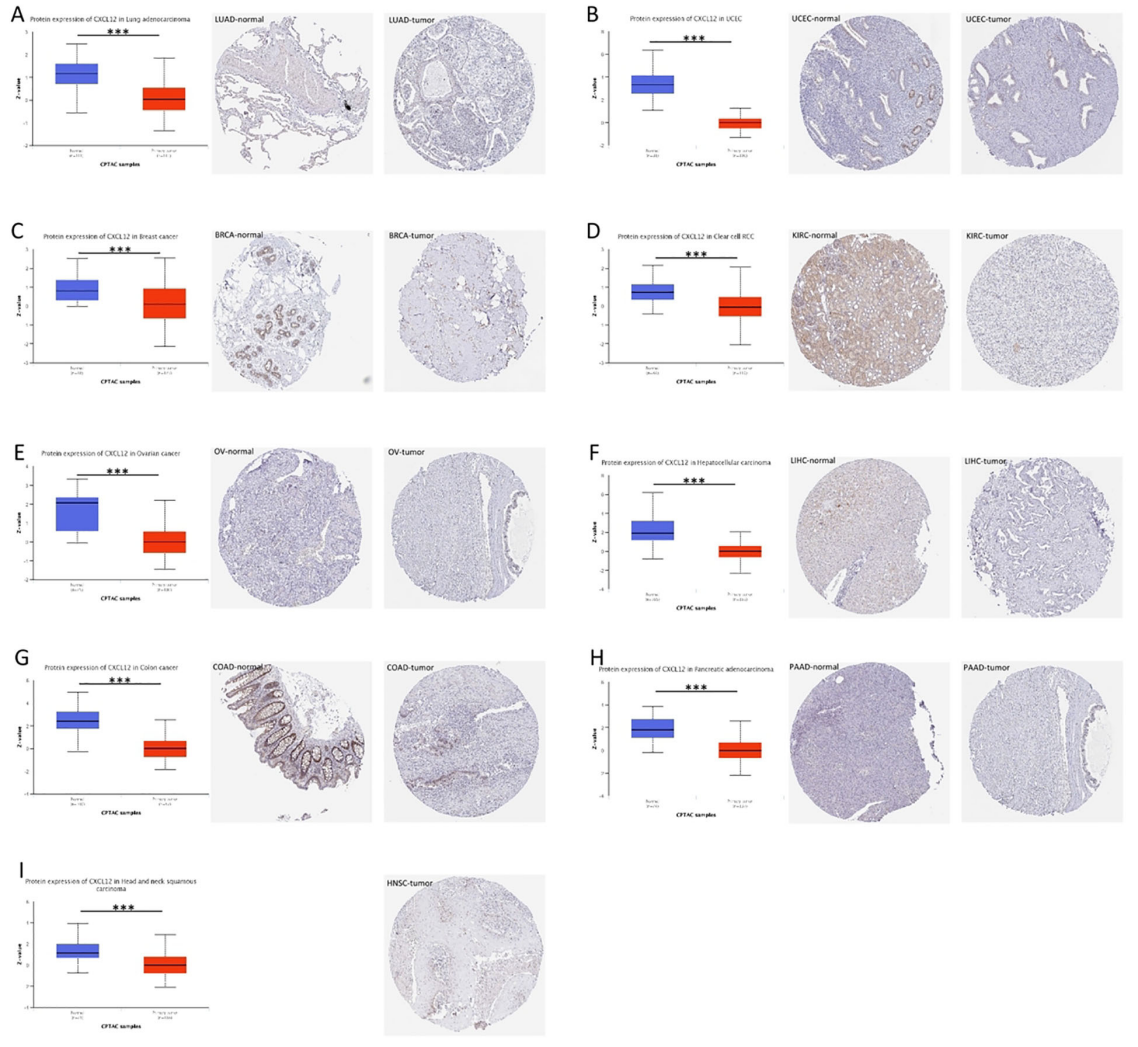
Expression of CXCL12 in tumor tissues and normal tissues of different cancers in TCGA database (left), immunohistochemical image of CXCL12 in normal tissues in HPA database (middle), and immunohistochemical image of CXCL12 in tumor tissues in HPA database (right). **(A)** LUAD; **(B)** UCEC; **(C)** BRCA; **(D)** KIRC; **(E)** OV; **(F)** LIHC; **(G)** COAD; **(H)** PAAD; **(I)** HNSC. (*P<0.05, **P<0.01, ***P<0.001).

The CXCL12 protein expression is reduced in LUAD, UCEC,BRCA, KIRC, OV, LIHC, COAD, PAAD, HNSC compared to normal tissue (**Figure 4**).

### The expression of CXCL12 in different stage

**Figure 5:**
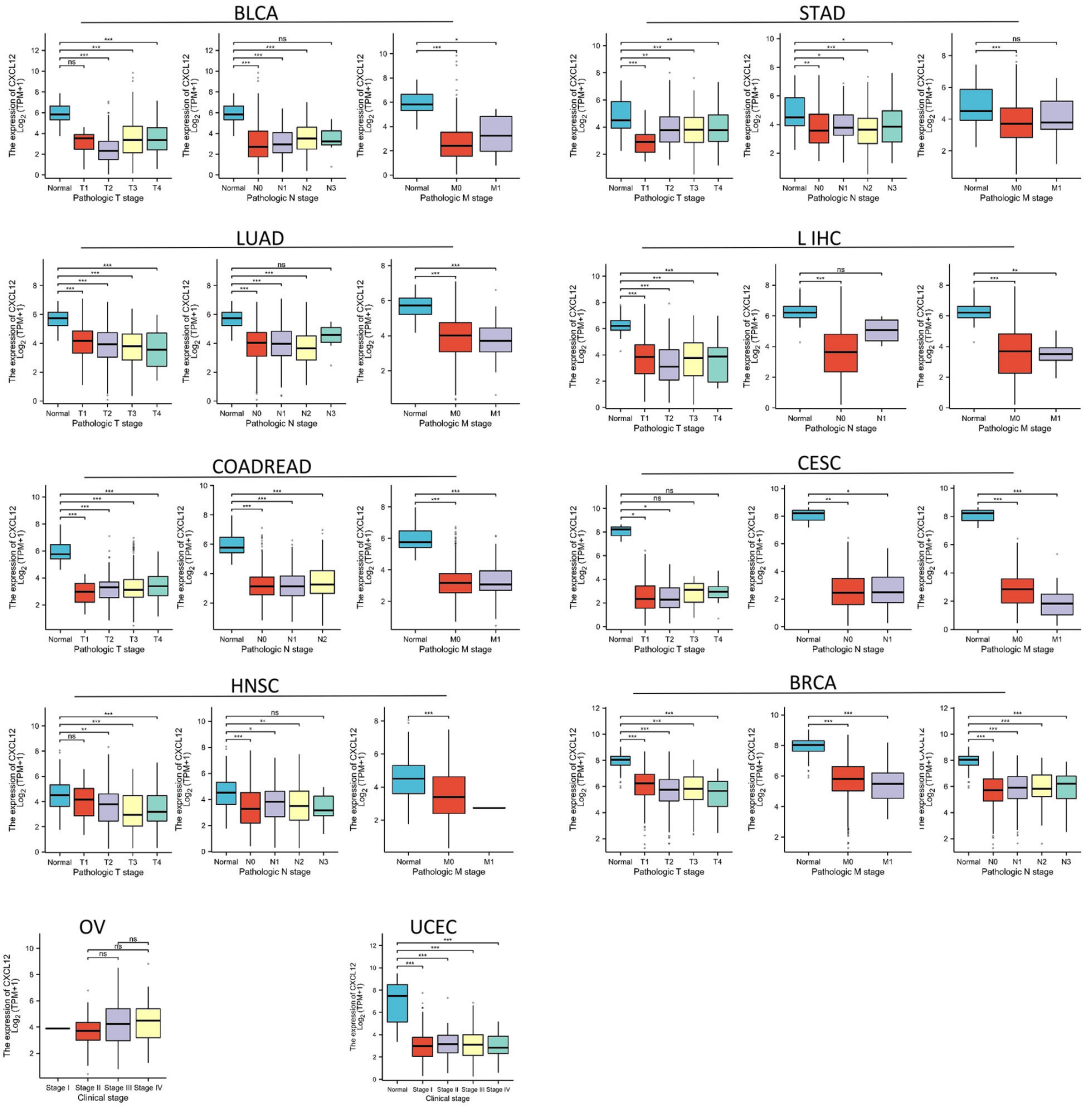
Association between CXCL12 expression and tumor stage. (*p < 0.05, **p < 0.01, ***p < 0.001. ns, not statistically significant).

CXCL12 expression reduces in initial stages of tumor during our study on its staging correlation, including BLCA, STAD, LUAD, LIHC, COADREAD, CESC,HNSC, BRCA, OV and UCEC indicating that CXCL12 potentially offers substantial clinical utility for diagnosing these tumors at an early stage (**Figure 5**).

### The diagnostic value of CXCL12 in the 33 cancers ROC

**Figure 6:**
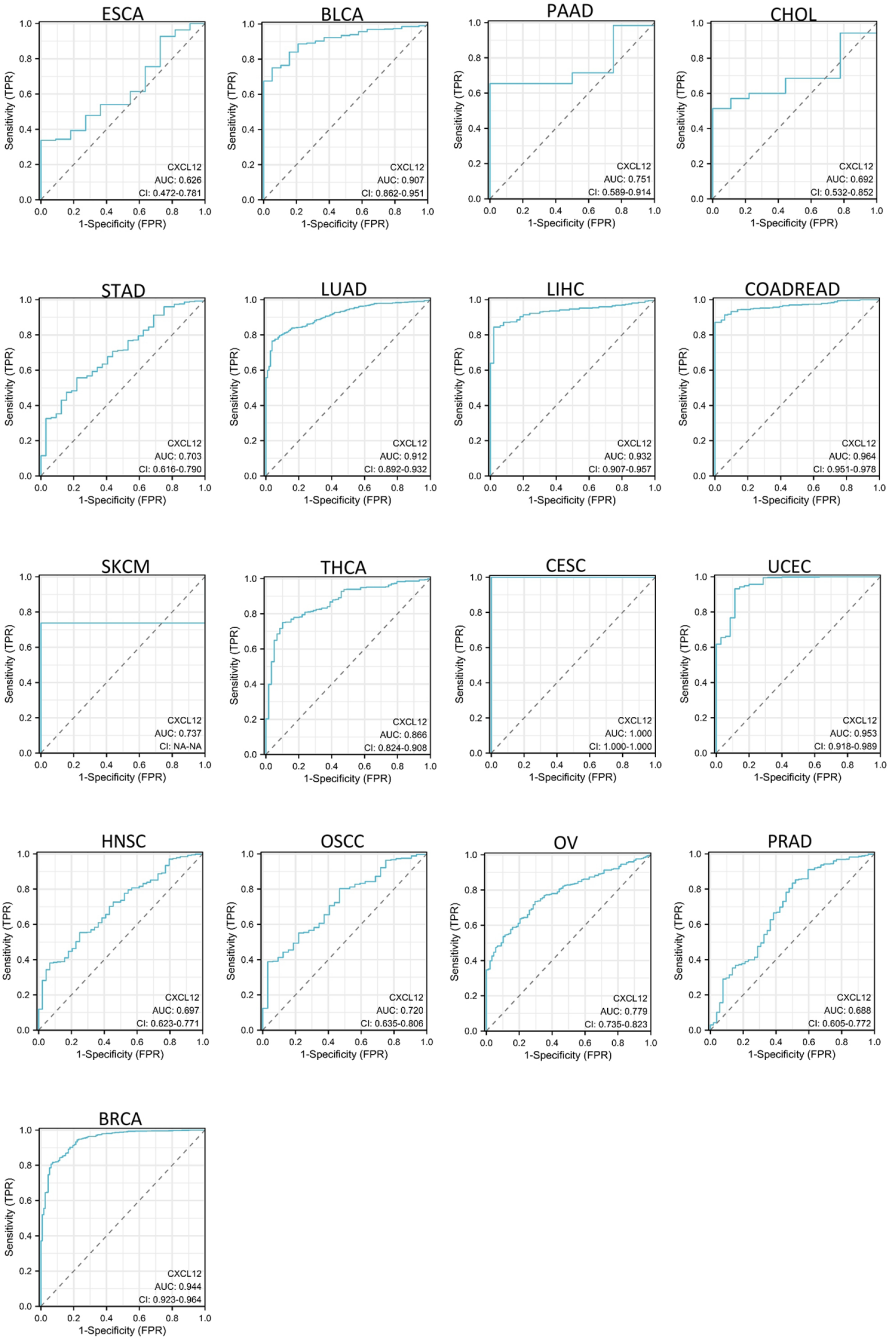
AUC of ROC curves verified the diagnosis performance of CXCL12 in the TCGA cohort.

As shown in **Figures 6**, CXCL12 has a good diagnostic value in a variety of cancers. Its AUC was greater than 0.7 in 13 cancers and even exceeded 0.9 in 7 cancers including CESC (AUC = 1), BLCA (AUC = 0.907), LUAD (AUC = 0.912),LIHC (AUC = 0.932), COADREAD (AUC = 0.964), UCEC(AUC = 0.953), BRCA (AUC = 0.944), PAAD (AUC = 0.715), STAD (AUC = 0.703), SKCM (AUC =0.737), THCA (AUC = 0.866), OSCC(AUC = 0.722), OV (AUC = 0.779), ESCA (AUC =0.626),CHOL(AUC = 0.692),HNSC (AUC = 0.697),PRAD (AUC = 0.688) (**Figure 6**).

### Survival analysis of CXCL12 in the 33 cancers OS

**Figure 7:**
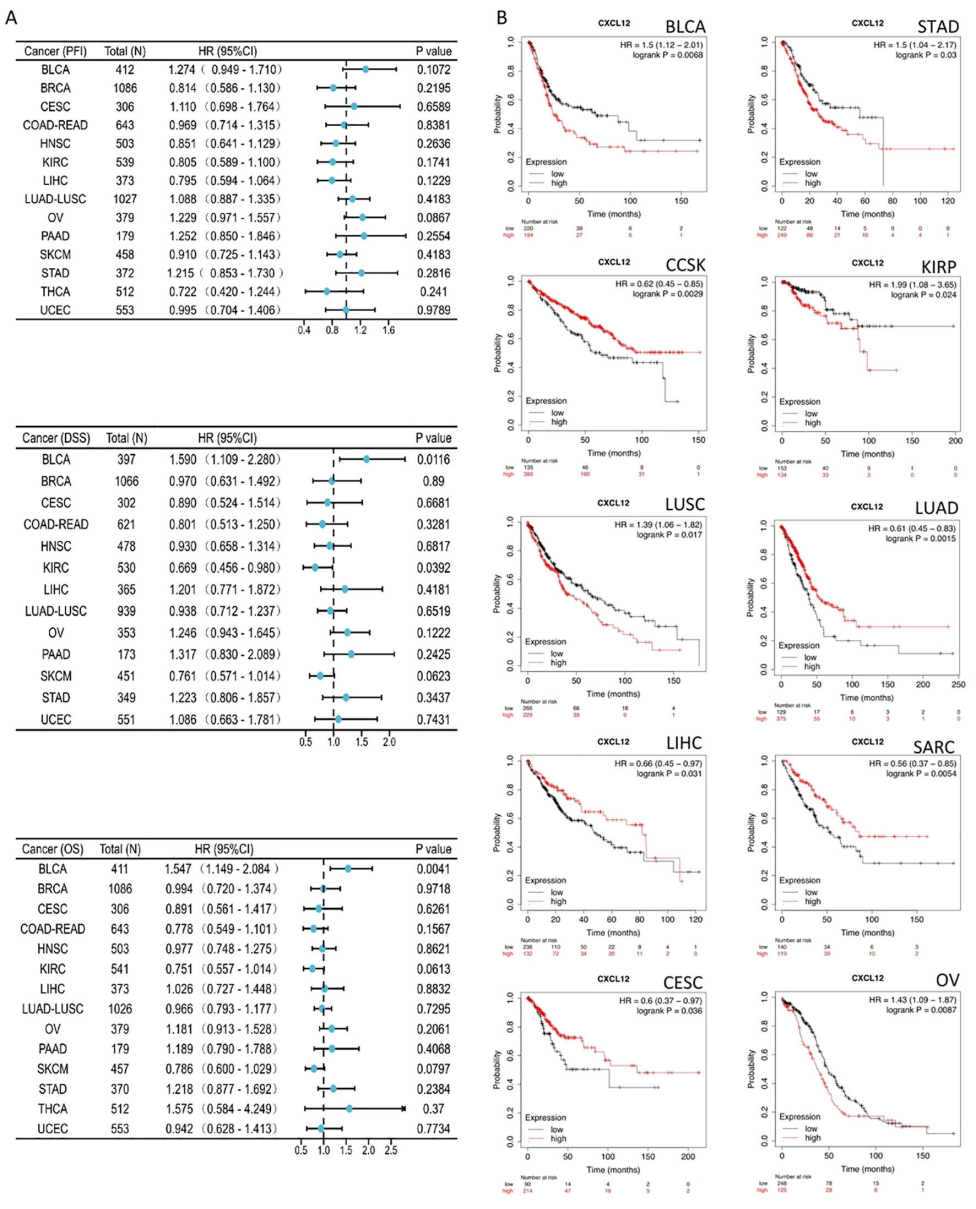
Association between CXCL12 expression and overall survival (OS). **(A)** Forest plot of PFI,DSS,OS associations in 33 types of tumor. **(B)** Kaplan-Meier analysis of the association between CXCL12 expression and OS.

The results suggest that for BLCA’s 33 cancers, CXCL12 acts as a significant risk factor for DSS.The result found that high CXCL12 groups have statistically better OS than those for the low CXCL12 groups in CCSK, LUAD, LIHC, LARC, CESE. However, the low CXCL12 groups show statistically better OS than high CXCL12 groups in BLCA, STAD, KIRP, LUSC, OV (**Figure 7**).

### CXCL12 expression in different immune and molecular subtypes of the 33 cancers

**Figure 8:**
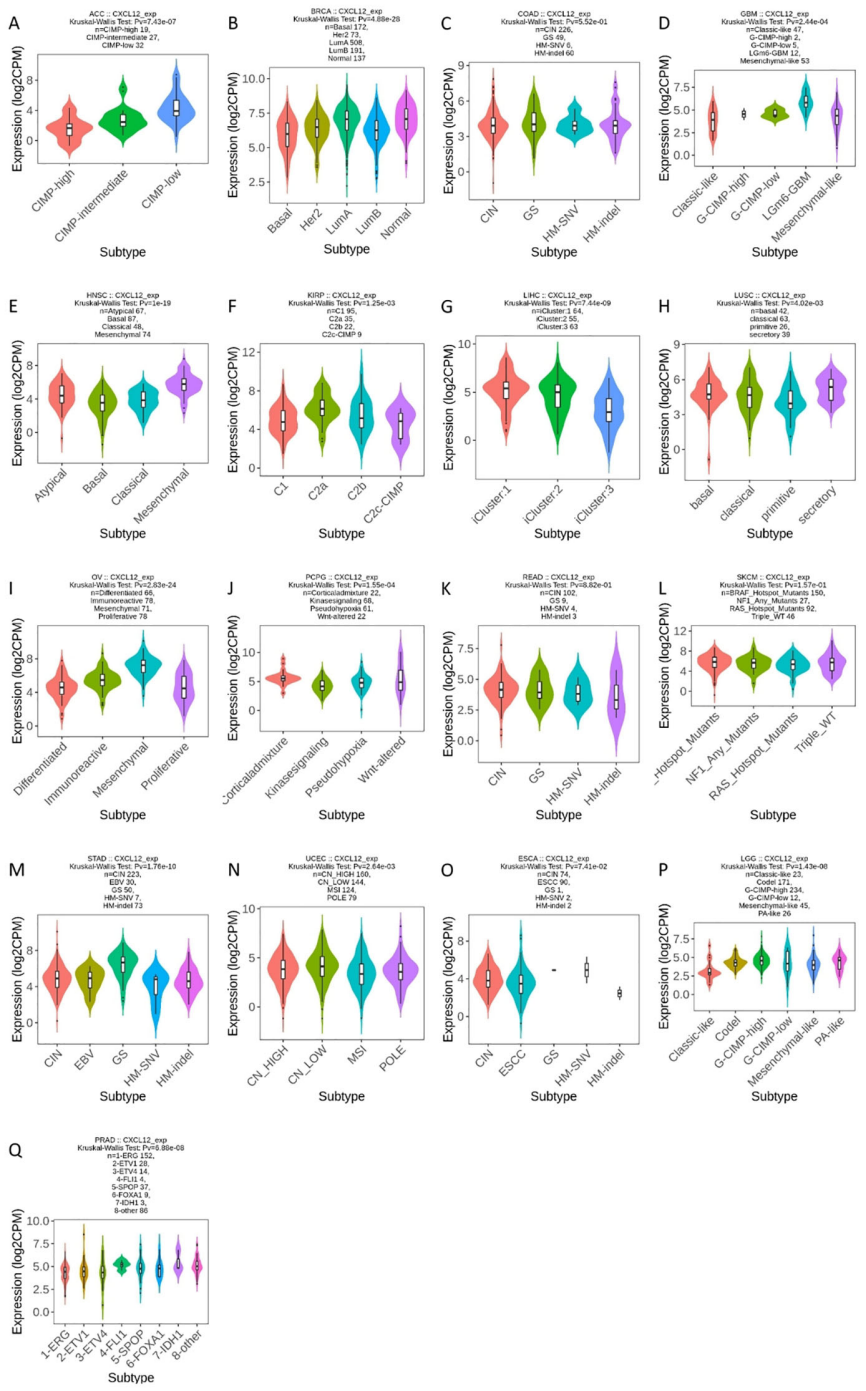
Correlations between CXCL12 expression and molecular subtypes in 17 cancers. **(A)** CIMP, CpG Island Methylator Phenotype; **(B)** Basal: ER-PR-Her2-, Her2: Her2+, LumA: ER+, LunB: ER+Her2+; **(C)** CIN, Chromosomal Instability; GS, Genomically Stable; HM-SNV, Hypermutated-Single Nucleotide Variant; HM-indel, Hypermutated-Insertion Deletion; **(D)** G- CIMP, Glioma-CpG island methylator phenotype; **(E)** HNSC, Head and Neck Squamous Cell Carcinoma; **(F)** KIRP, Kidney Papillary Rell Rarcinoma; **(G)** LIHC, Liver Hepatocellular Carcinoma; **(H)** LUSC, Lung Squamous Cell Carcinoma; **(I)** OV, Ovarian Cancer; **(J)** PCPG, Pheochromocytoma and Paraganglioma; **(K)** READ, Rectum Adenocarcinoma; CIN, Chromosomal Instability; GS, Genomically Stable; HM-SNV, Hypermutated-Single Nucleotide Variant; HM-INDEL, Hypermutated-Insertion Deletion; **(L)** SKCM, Skin Cutaneous Melanoma; **(M)** STAD, Stomach Adenocarcinoma; CIN, Chromosomal Instability; EBV, Epstein-Barr Virus; GS, Genomically Stable; HM-SNV, Hypermutated-Single Nucleotide Variant; HM-INDel, Hypermutated-Insertion Deletion; **(N)** UCEC, Uterine Corpus Endometrial Carcinoma; CN-HIGH, Copy Number High; CN-LOW, Copy Number Low; MSI, Microsatellite Instability; POLE, Polymerase Epsilon; **(O)** ESCA, Esophageal Squamous Cell Carcinoma; CIN, Chromosomal Instability; ESCC, Esophageal Squamous Cell Carcinoma; GS, Genomically Stable; HM-SNV, Hypermutated-Single Nucleotide Variant; HM-INDEL, Hypermutated-Insertion Deletion; **(P)** LGG, Lower Grade Glioma; G-CIMP, Glioma CpG Island Methylator Phenotype; **(Q)** PRAD, Prostate Adenocarcinoma; ERG, Erythroblast Transformation-Specific Gene.

**Figure 9:**
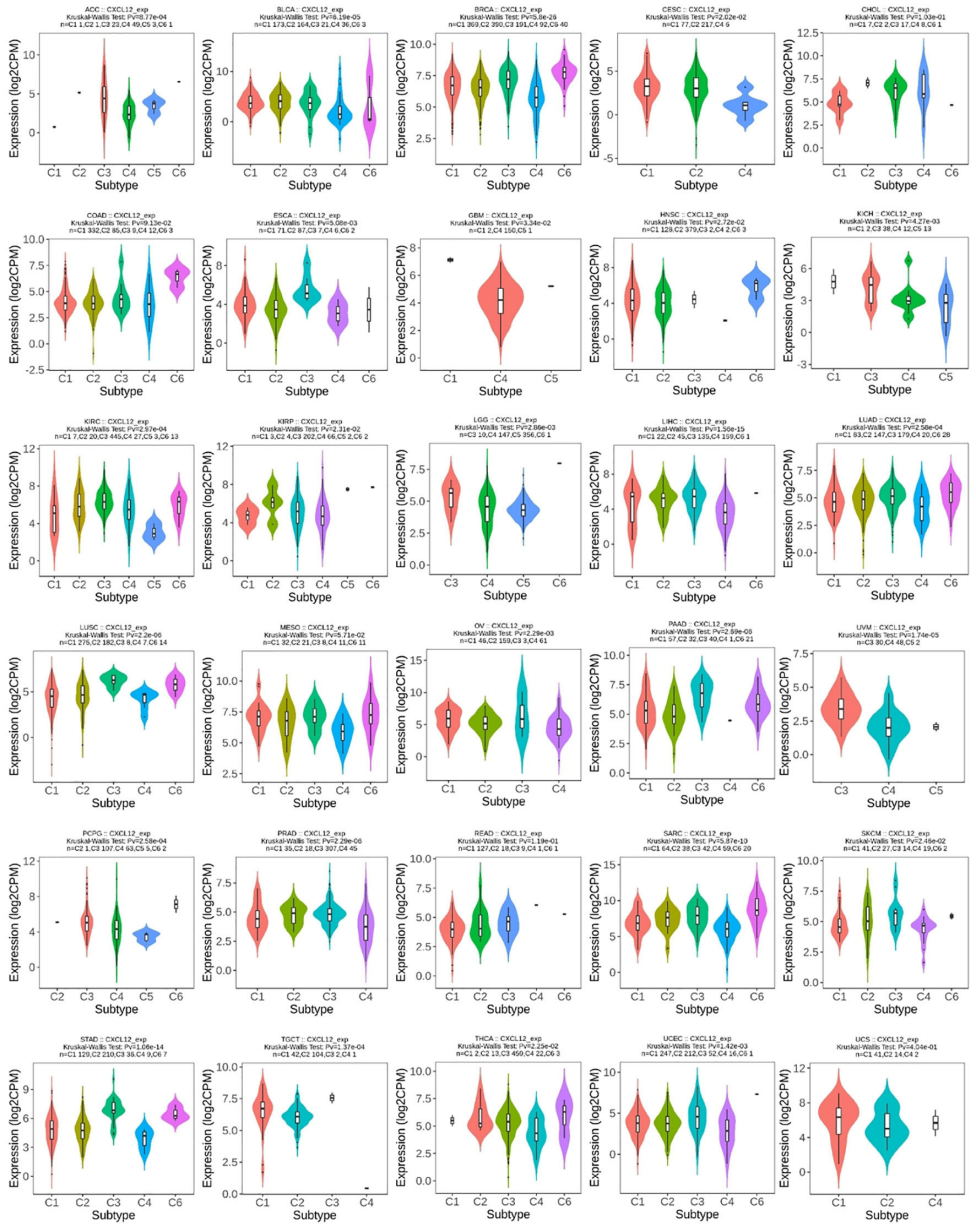
Correlations between CXCL12 expression and immune subtypes in 30 cancers. C1 (wound healing), C2 (IFN-g dominant), C3 (inflammatory), C4 (lymphocyte deplete), C5 (immunologically quiet), and C6 (TGF-b dominant).

For molecular subtypes, CXCL12 expresses significantly differently in 13 cancer types, including ACC, BRCA, GBM, HNSC, KIRP, LIHC, LUSC, OV, PCPG, STAD, UCEC, LGG, PRAD (**Figure 8**). Results indicate significant divergence between immune subtypes in ACC (six subtypes), BLCA (six subtypes), BRCA (six subtypes), CESC (three subtypes), ESCA (six subtypes), GBM (three subtypes), HNSC (six subtypes), KICH (four subtypes), KILC (six subtypes), KIRP (six subtypes), LGG (four subtypes), LICH (five subtypes), LUAD (five subtypes), lusc (five subtypes), OV (four subtypes), PAAD (five subtypes), UVM (three subtypes), PCPG (five subtypes), PRAD (four subtypes), SARC (five subtypes), SKCM (five subtypes), STAD (five subtypes), tgct (four subtypes), THCA (five subtypes), UCEC (five subtypes) (**Figure 9**).

### Genetic mutations of CXCL12

**Figure 10:**
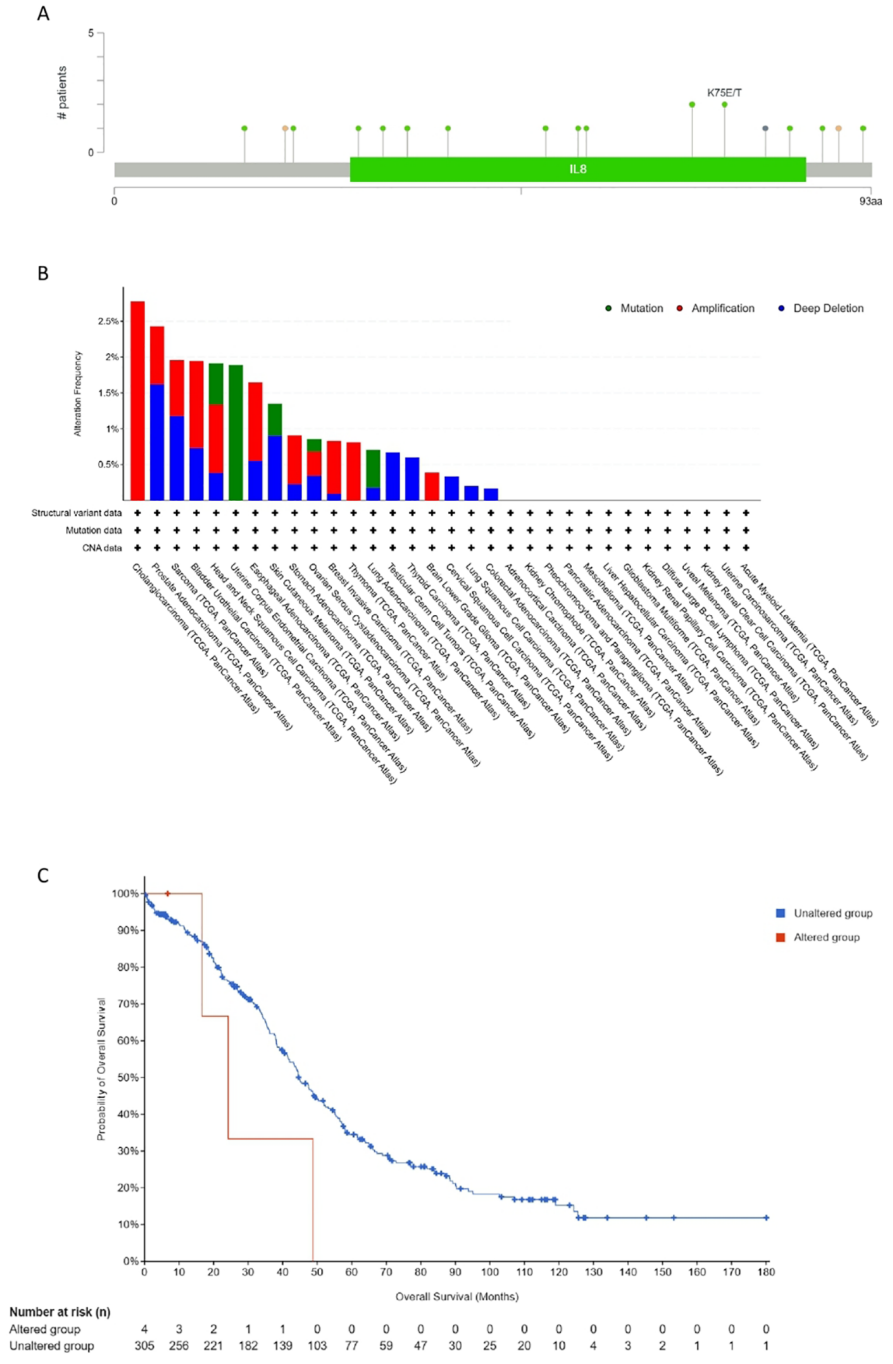
Mutation feature of CXCL12 in different tumors of TCGA. We analyzed the mutation features of CXCL12 for the TCGA tumors using the cBioPortal tool. The alteration frequency with mutation site **(A)** and mutation type **(B)** are displayed. **(C)** We also analyzed the potential correlation between mutation status and overall survival of OV using the cBioPortal tool.

A total of 17 sites from amino acid positions 0 to 93 exhibited mutation activity, including 13 missense, two truncation, one splice, one fusion (SV) and most predominantly, K75E/T (Missense). The predominant mutation categories identified included Missense, Amplification, and Deep Deletion. Notably, CXCL12’s gene alteration across various tumor tissues was assessed using this platform; amplifications being the most prevalent. Mutations of CXCL12 notably occurred more frequently in HNSC and UCEC. Deep deletions were prevalent in PRAC, Sarcoma, and BRAC among the 32 cancers examined. Furthermore, an investigation into the correlation between CXCL12 genetic alternations and clinical survival outcomes in ovarian cancer revealed poorer prognoses amongst patients with altered CXCL12 (**Figure 10**).

### Promoter methylation levels of CXCL12

**Figure 11:**
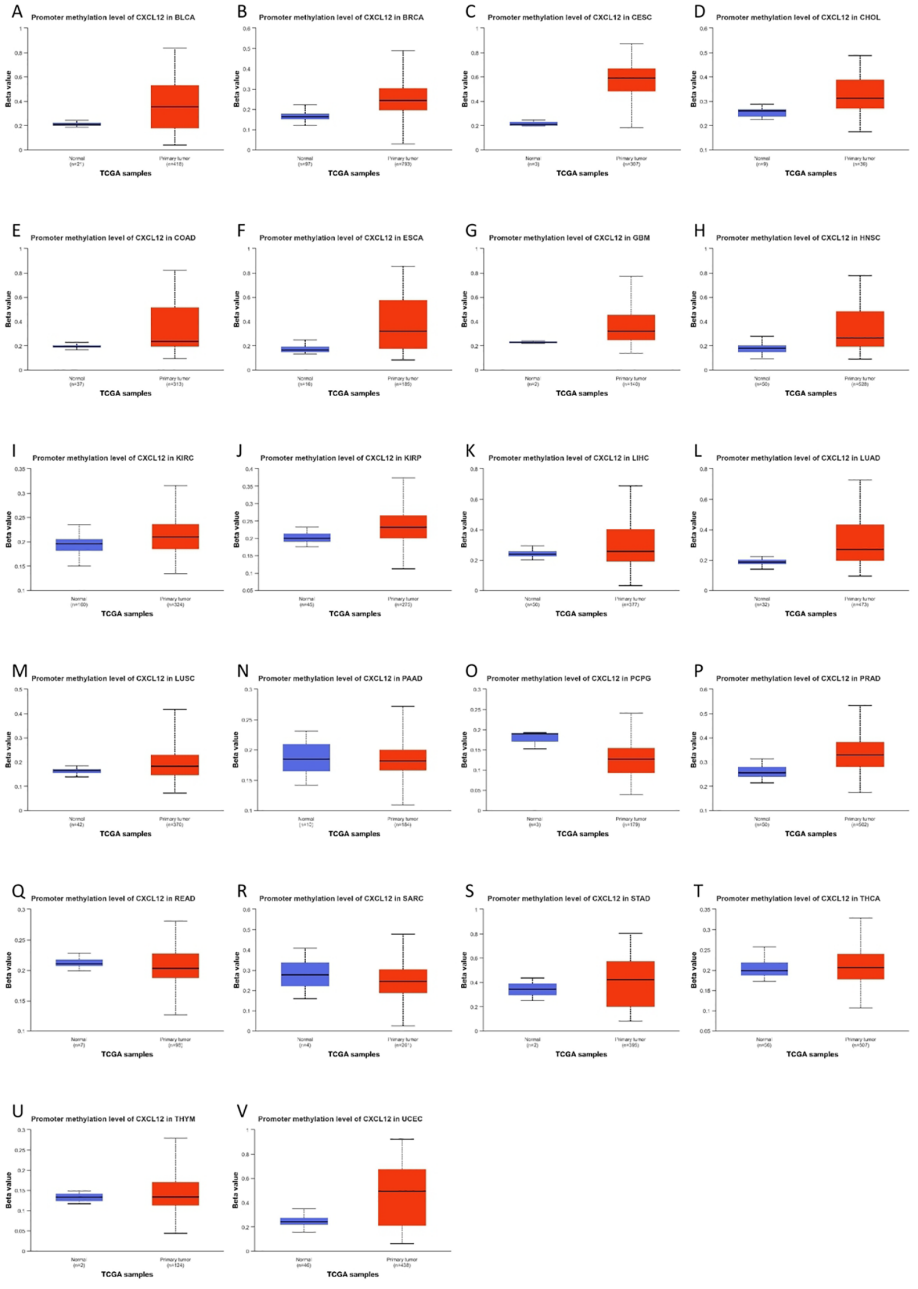
The promoter methylation level of CXCL12 in cancers.

**Figure 11** illustrates elevated CXCL12 promoter methylation in 17 tumor groups compared to controls (**Figure 11**).

### The PPI, functional enrichment of CXCL12 in cancers

**Figure 12:**
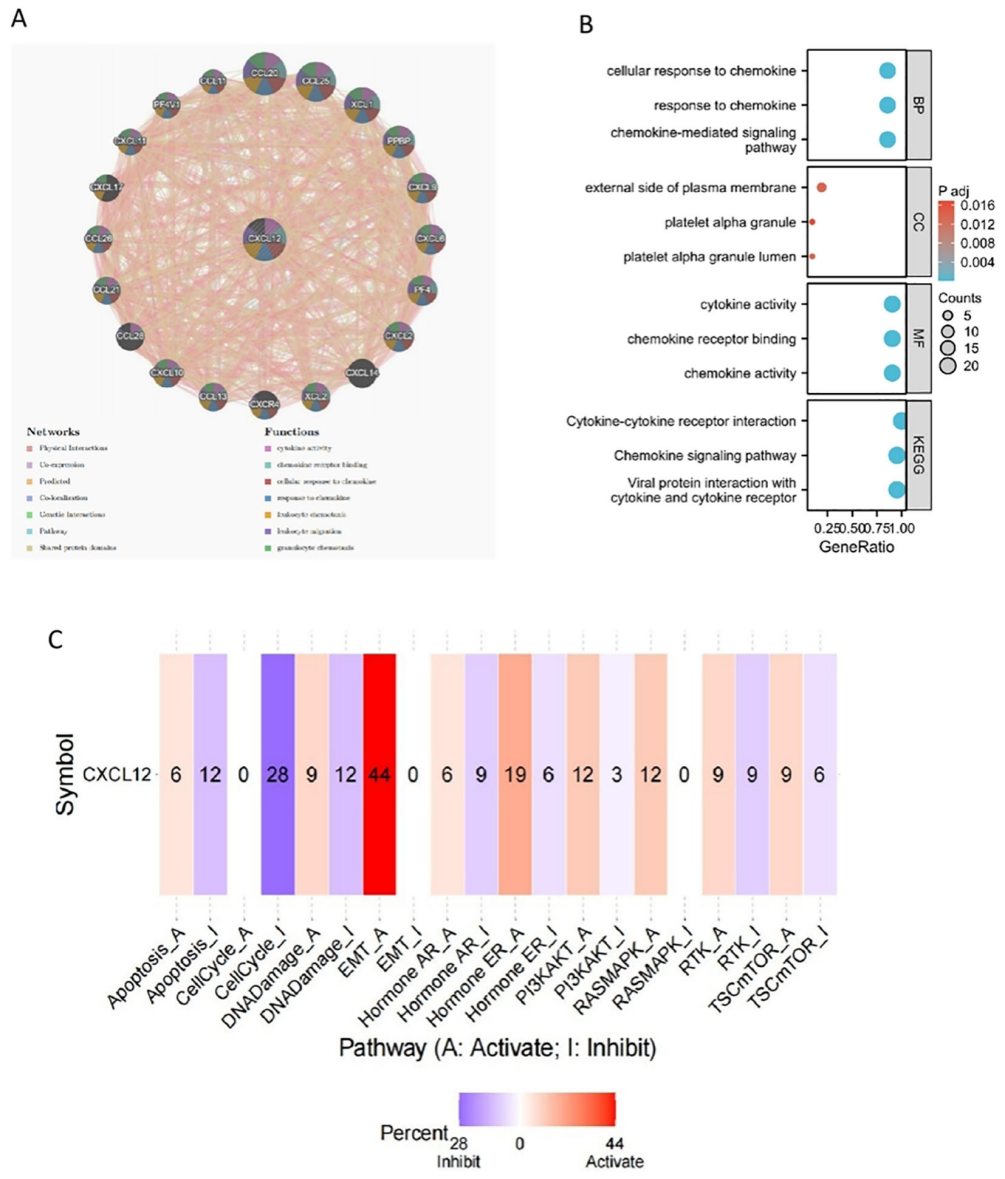
The gene-gene interaction network of CXCL12 from GeneMANIA;**(B)** GO/KEGG pathway enrichment for CXCL12 and closed interact genes; **(C)** CXCL12 with pathway activity or inhibition.

We identified 21 genes in tight correlation with CXCL12, forming a PPI network. Analysis revealed GO/KEGG enrichments among these genes. Three major classifications under RNA function include biological process (BP), molecular function (MF), and cellular component (CC). Key GO terms relevant to BP include chemokine signaling pathway, cell response to chemokines, and response to chemokines. For CC, they encompass external side of plasma membrane, platelet alpha granule lumen, and platelet alpha granule. As for MF, they involve chemokine activity, chemokine receptor binding, and cytokine activity. Top KEGG pathways include Viral protein interaction with cytokine and cytokine receptor, Chemokine signaling pathway, and Cytokine-cytokine receptor interaction. Pathways activated by CXCL12, predominantly in cancer cells, include EMT, hormone ER, PI3K/AKT, RAS/MAPK, RTK, and TSC/mTOR. Conversely, apoptosis, cell cycle, DNA damage, hormone AR, and RTK are suppressed (**Figure 12**).

### Functional states of CXCL12 in scRNA-Seq datasets

**Figure 13:**
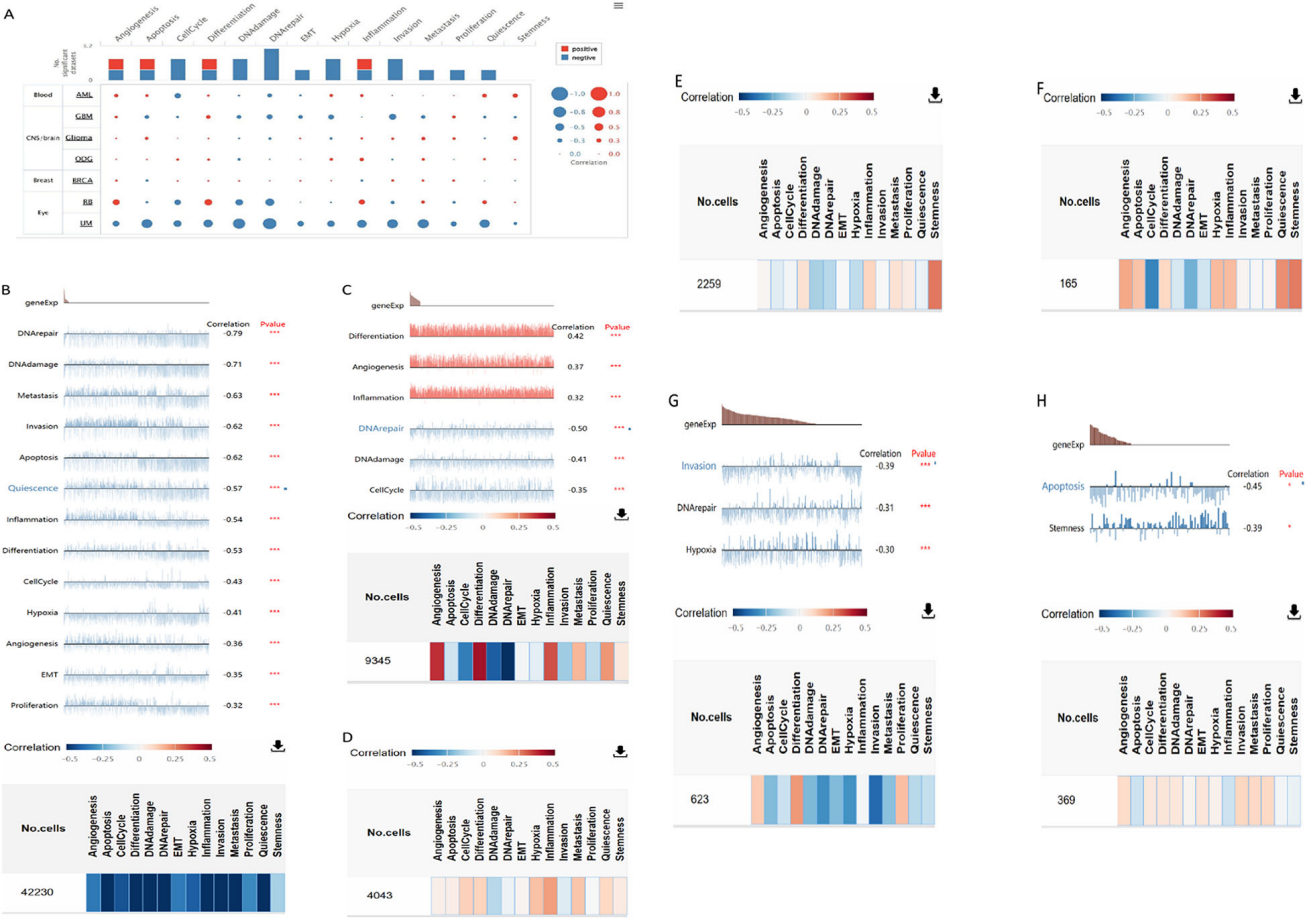
The correlation of CXCL12 with functional state in cancers. **(A)** The interactive bubble chart present correlation of CXCL12 with functional state in76 cancers; The correlation of CXCL12 with functional state in **(B)** UM, **(C)**RB, **(D)** ODG, **(E)** Glioma, **(F)** AML, **(G)** GBM, **(H)**BRCA. (***p < 0.001, **p < 0.01, *p < 0.05).

Negative relationships between CXCL12 and key biological processes such as cell cycle, DNA damage/repair, epithelial-to-mesenchymal transition (EMT), hypoxia, invasion, metastasis, proliferation, and quiescence suggested potential tumor suppressor roles for CXCL12. Examining CXCL12’s correlation with specific cancer functions revealed positive links with inflammation, differentiation, and angiogenesis in retinoblastoma (RB) but negative correlations with DNA repair, DNA damage, and cell cycle. In osteosarcoma (OG), CXCL12 was positively associated with inflammation, metastasis, cell cycle, differentiation, hypoxia, and negatively related to DNA repair, DNA damage, and invasion (**Figure 13**).

### Immunogenomic analyses of CXCL12 in the 33 cancers

**Figure 14:**
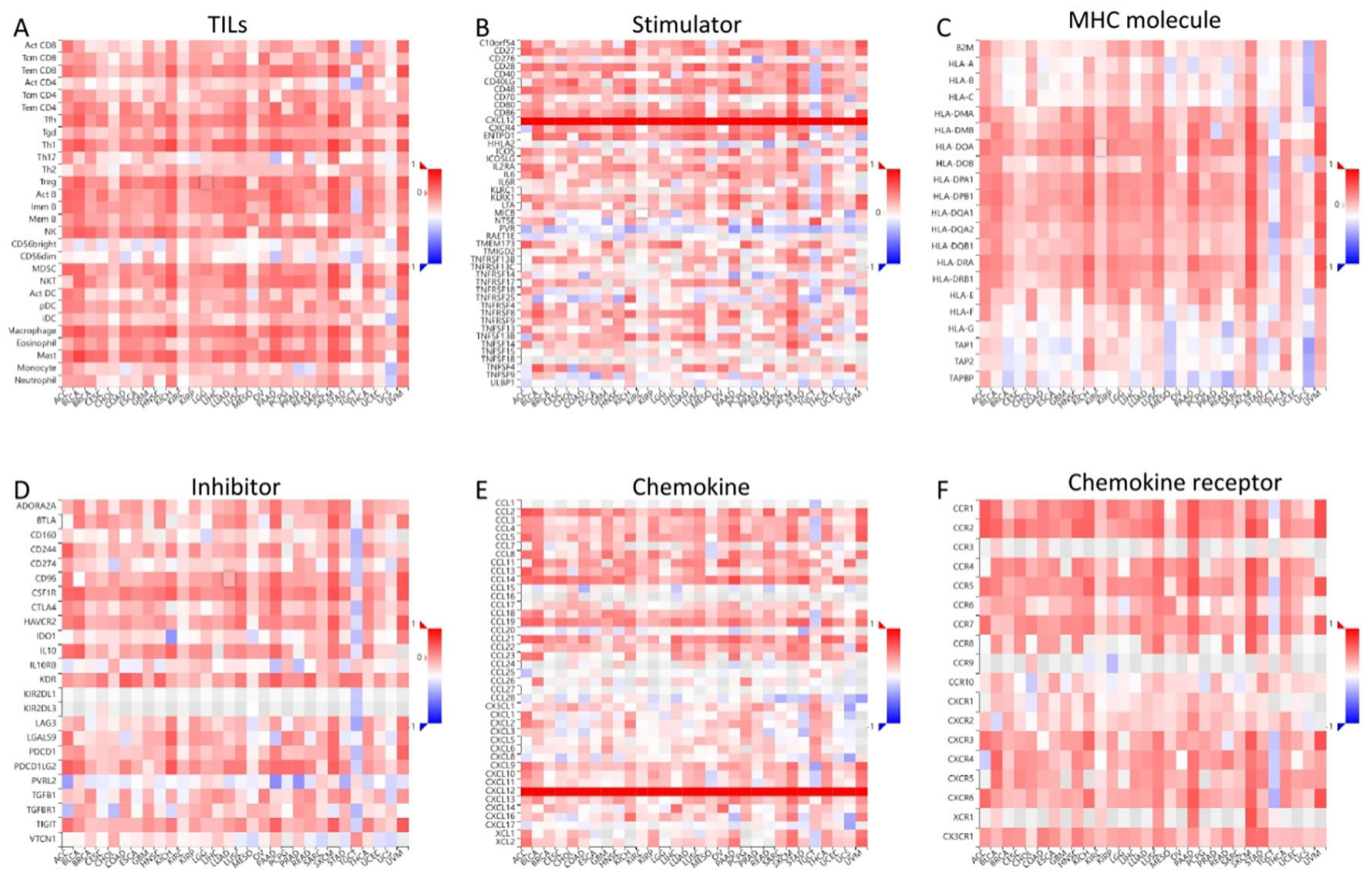
Correlation of CXCL12 with TILs and immunoregulation-related genes in 33 cancers. Correlations between CXCL12 expression and **(A)** TILs, **(B)**Immunostimulators, **(C)** MHC molecules, **(D)** Immunoinhibitors, **(E)** Chemokines, **(F)** Chemokine receptors. (*p < 0.05, **p < 0.01).

**Figure 15:**
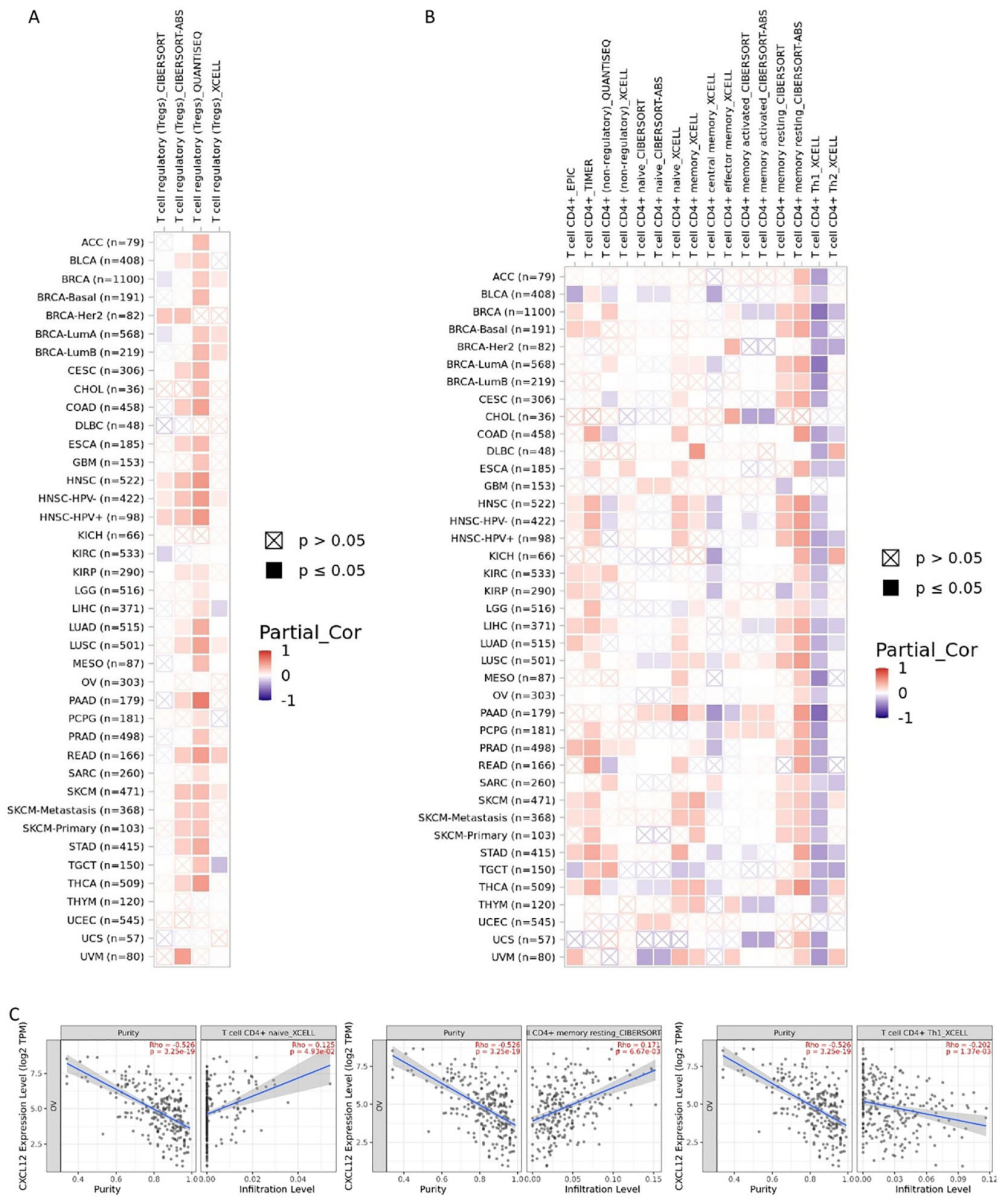
Correlation analysis between CXCL12 expression and immune infiltration of Treg cell **(A)** and CD4+ T cell **(B)** across all types of cancer in TCGA. Different algorithms were used to explore the potential correlation between the expression level of the CXCL12 gene and the infiltration level of Treg cell and CD4+ T cell in OV **(C)**.

Results indicated a significant positive correlation between CXCL12 and most immune cells across 33 cancers, notably SKCM, KICH, UVM. Notably, CXCL12 displayed a positive correlation with most immunostimulators, excluding KIR2DL1 and KIR2DL3, specifically in HNSC, KIRC, KIRP, LIHC, STAD, THCA, and UVM. A positive correlation with most MHCs was observed in KICH, UVM, SKCM, LUSC, CHOL, while a negative correlation with most MHCs was seen in UCS and STAD. Positive correlations were found between CXCL12 and most cytokines in COAD, KIRC, LGG, LIHC, PRAD, THCA, and UVM. Concerning cytokine receptors, CXCL12 exhibited a positive correlation with most in HNSC, KIRC, LIHC, PRAD, THCA. Utilizing TIMER, CIBERSORT, CIBERSORT-ABS, QUANTISEQ, XCELL, MCPCOUNTER, and EPIC algorithms, we examined the potential link between CXCL12 expression and immune cell infiltration in various TCGA cancer types. Analysis revealed a statistically significant positive correlation between Treg cell infiltration and CXCL12 expression in HNSC, READ, SKCM using multiple algorithms. Conversely, a negative correlation was observed between Th1 cell infiltration and CXCL12 expression in most tumor types. Scatterplot data demonstrated a positive correlation between CXCL12 expression in OV and infiltration by T cell CD4+ naive and memory as per the XCELL and CIBERSORT algorithm, and a negative correlation with T cell CD4+ Th1 infiltration as per the XCELL algorithm (**Figures 14**, **15**).

### Immunohistochemical

**Figure 16:**
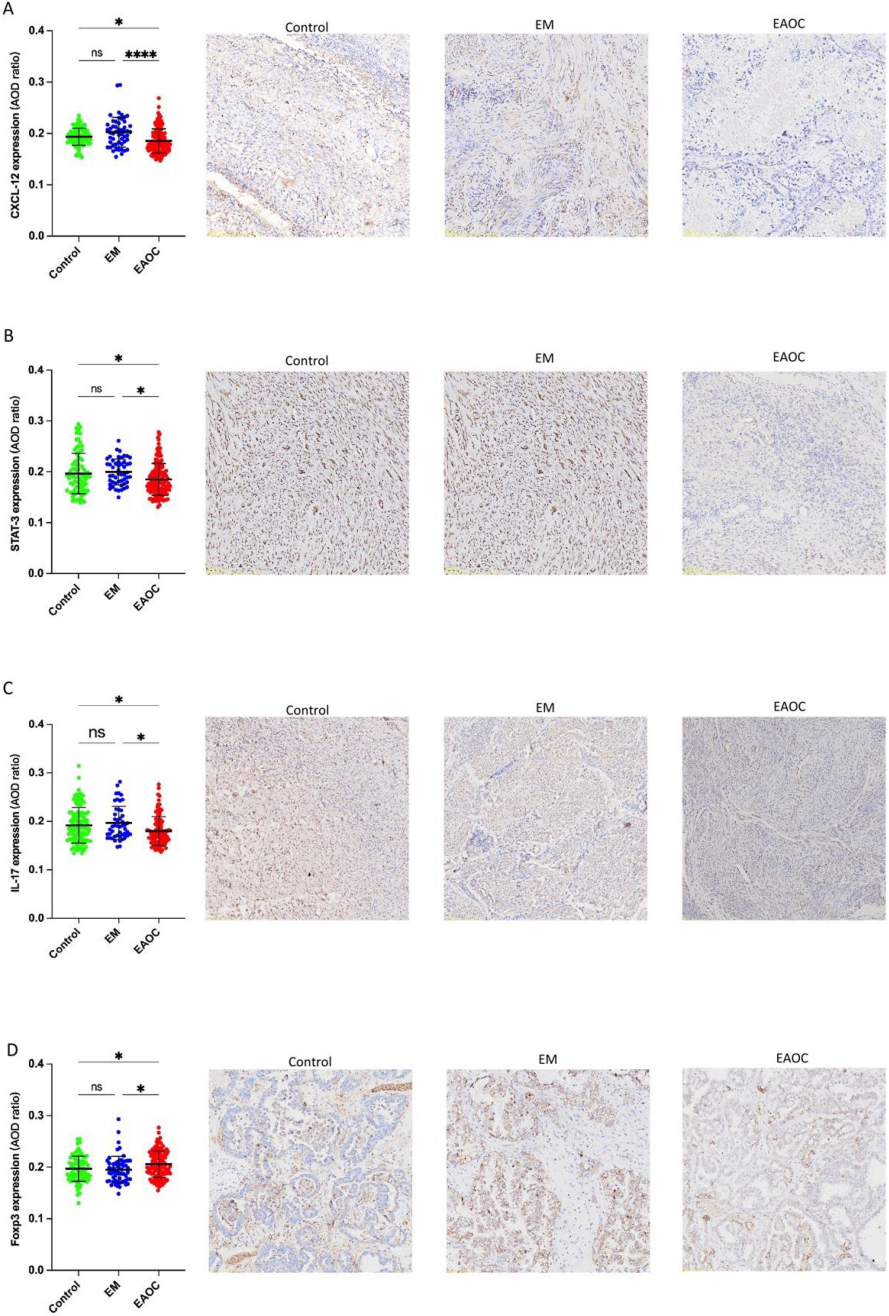
Expression of CXCL12 **(A)**; STAT3 **(B)**; IL-17 **(C)**; FOXP3 **(D)** in EAOC, EM and normal endometrium tissues.

Immunohistochemistry revealed markedly decreased CXCL12, IL17, STAT3 levels and elevated FOXP3 expression in 26 EAOC samples compared with both EM and control endometrial tissue (**Figure 16**). Moreover, a reduced Th17/Treg ratio was seen in EAOC (1.045 ± 0.119) as opposed to EM (0.951 ± 0.100) and controls (0.934 ± 0.143).

## Discussion

Endometriosis, a women’s reproductive age disorder with ectopic endometrium growth, is linked to elevated rates of some malignancies, notably EAOC. Although uncommon, ovarian cancer incidence escalates among endometriosis sufferers, primarily for endometrioid and clear-cell types (13, 35). Autophagy is an essential process in cells, responsible for the recycling and degradation of damaged organelles or unwanted macromolecular complexes. It’s a highly conserved catabolic pathway evolutionarily speaking (36). There’s a complex link between endometriosis and ovarian cancer, with autophagy playing crucial roles in both conditions’ progression. It’s involved not just in endometriosis but also impacts ovarian cancer cells’ behavior, potentially aiding the latter’s growth through influences such as drug resistance, dormancy maintenance, or stem cell properties preservation (37). Hence, delving into the role and mechanism of autophagy in ovarian cancers linked to endometriosis bears significance in understanding these two diseases’ connection and devising novel treatments.

GEO, Networkanalyst, and autophagy-specific genes identification were employed to detect significant autophagy-associated DEGs in endometriosis versus EAOC. Our results indicate lower CXCL12 expression in EAOC patients, distinct from endometriosis.

Indeed, chemokines, a category of small, protein molecules exerting chemoattractant effects, are synthesized by tumorigenic and stromal cells, which can be regulated by chemical inducers and cytokines to stimulate the chemotactic motility of cells. Based on the position of cysteine (C) residues, chemokines can be classified into four categories: CXCCCC and CXC3. Cytokine receptors, part of the G protein-coupled receptor superfamily(GPCRS) and are classified into CXCRCCR, CR and CX3CR based on their ligand specificity (38). Aberrant expression of CXCL12/CXCR4 has been observed in diverse tumor types, and are believed to act as a crucial chemokine-chemokine receptor in promoting tumor growth.CXCL12 and CXCR4 have direct or indirect effects on tumor development. On one hand, CXCL12/CXCR4 stimulation initiates multiple signaling cascades regulating Ca2+ flux, migration transcription, and cellular viability (39). On the other hand, they affect tumor development through direct or indirect effects. The key role of CXCL12 and its receptor, CXCR4, is in regulating the accumulation of stromal and immune cells within the microenvironment (TME).This process leads to the formation of a specific immune microenvironment that significantly impacts tumor growth, invasion, metastasis, angiogenesis, and drug resistance (40). The gene CXCL12, also recognized as stromal cell-derived factor 1 (SDF-1), resides on chromosome 10q11 of the human genome. It is widely expressed in different tissues and plays a crucial role in embryogenesis, angiogenesis, immune cell generation and recruitment of stem cells (41). CXCL12 has the ability to bind to both CXCR4 and CXCR7 receptors. Bioinformatic scrutiny revealed the pivotal role of CXCL12 in pan-cancer. Our research assessed CXCL12 mRNA and protein levels in diverse human specimens, critically comparing these to cancerous states. We evaluated the diagnostic and predictive implications of CXCL12 expression in varied forms of cancer, together with delineating its variation in various immune cell subsets therein. CXCL12’s predominant variants and corresponding sites were pinpointed. PPI regulatory networks for CXCL12 were strategically designed, investigating its activation/inhibition mechanisms, revealing its enriched functional pathways, and assessing its activity at a cellular level in select cancers. Lastly, a correlation study was performed examining CXCL12 expression and immune cell infiltration in multiple cancer types.

Cancer research is a prevailing theme in contemporary medicine. We used 33 cancer datasets from TCGA and CCLE platforms to uncover potential biomarkers for comprehensive cancer diagnosis via gene expression disparity analysis. The primary focus was on CXCL12, examined comprehensively within multiple cancers. This pan-cancer analysis revealed its substantial downregulation in numerous cancer types and its differing expression levels between malignant and healthy tissue. Its useful application for early detection, regulatory mechanisms, and associated genes were also discussed. Emerging evidence suggests a correlation between CXCL12 and disease states, notably tumors. The precise role of CXCL12 in tumorigenesis through shared molecular pathways requires further exploration. To our knowledge, no prior studies have conducted a pan-cancer analysis of CXCL12 across diverse tumor types.

Using the GTEx and TPA datasets, we conducted an evaluation of CXCL12 gene/protein expression across a range of tissues. Our analysis revealed a significant upregulation in adipose tissue, endometrium, spleen, smooth muscle, while showing inhibition in placental and cerebral cortex tissues. Additionally, investigating the pan-cancer context via unpaired sample analysis in the TCGA-GTEx dataset revealed a downregulated CXCL12 expression in cancerous tissues (e.g. BLCA, BRCA, CESC, etc.) compared to their corresponding normal counterparts. Conversely, it was elevated in DLBC, LAML, LGG, TGCT. No significant alterations were observed in GBM, PAAD, PCPG, or their corresponding normal tissues. Moving to the protein level, we conducted a thorough analysis of the CPTAC and HPA immunohistochemistry data, which indicated diminished CXCL12 protein expression in LUAD, UCEC, BRCA, KIRC, OV, LIHC, COAD, PAAD, HNSC. Furthermore, our analysis of the correlation between tumor stage and CXCL12 expression revealed a progressive decrease in CXCL12 expression in early stages (e.g. BLCA, STAD, LUAD, etc.) suggesting its potential role in early tumor detection.

With the advancement of research in tumor immunology, the concept of ‘tumor microenvironment’ has been introduced (42). This complex network consists of diverse cells, matrix surroundings, cytokines, vessels and lymphatic ducts, as well as tumor cell analysis and related physical factors. Together, these components shape the internal terrain of a tumor (43). This dynamic environment significantly impacts tumor initiation, progression, and metastasis. It has been reported that immune imbalance and chronic inflammation are significant contributors to cancer development (44). CD4+ T cells can differentiate into Th1, Th2, Treg, and Th17 subsets under different immune response stages, each with distinct biological functions. Th17 cells, a CD4+ T subset highly expressing interleukin-17 (IL-17), develop from naive CD4+ T cells via TGF-β and IL-6 stimulation. As potent proinflammatory cells, they activate dendritic cells and T helper lymphocytes, induce various cytokines, perpetuate chronic inflammation, and contribute to carcinogenic microenvironments (45). Additionally, Th17 cells secrete IL-17, TNF-α, and IL-21, with IL-17 being an essential proinflammatory cytokine, inducing expression of other chemokines, proinflammatory cytokines, and metalloproteinases, thereby exacerbating inflammatory cell infiltration and tissue damage (46). Treg cells, derived from naive CD4+ T cells independently by TGF-β,are immunosuppressive cells that regulate immune response intensity, mitigate immune injury, mediate immune evasion through anti-tumor immune suppression, and promote tumor progression (47). Under certain conditions, Treg and Th17 cells reciprocally transform to maintain immune system stability, playing crucial roles in anti-tumor immunity. A growing body of evidence suggests that Th17/Treg cell ratio imbalance promotes inflammation and tumor progression (48). Our analysis indicates that Th17/Treg cell balance is crucial in understanding endometriosis-associated ovarian cancer (EAOC), leading us to collect formalin-fixed, paraffin-embedded (FFPE) samples of EAOC, EM, and normal endometrium for immunohistochemistry evaluations of CXCL12, FOXP3, STAT3, and IL17.

Despite the finding that CXCL12 expression and the ratio of Th17/Treg correlates with patient in EAOC, we were unable to demonstrate a direct impact of CXCL12 on patient through immune infiltration. Future research, targeting CXCL12 expression and immune infiltrates within a cancer demographic, may clarify these findings.

Despite integration of multi-database information, certain limitations existed within this research. Firstly, extensive microarray and sequencing datasets analyzed primarily tumor tissue, potentially introducing bias during cell-level immune marker evaluations. To address this, high-resolution methodologies, like single cell RNA sequencing, are recommended. Secondly, lax attention was given to accurately representing CXCL12’s posttranslational modifications in these databases. Indeed, both phosphorylation and ubiquitination can alter CXCL12 functionality. Thirdly, conflicting data from disparate databases blur CXCL12’s clear classification. This study exclusively examined computational CXCL12 expression-survival correlations across databases, excluding *in vivo*/*in vitro* experimentation. Such studies aimed at elucidating CXCL12’s effects at cellular and molecular levels may assist understanding its role more effectively.

## Conclusions

Our comprehensive investigations linked CXCL12 expression to prognosis, DNA methylation, immune cell influx, genetic alterations, and microsatellite stability across various cancers. These results offer crucial insights into CXCL12’s role in cancer initiation utilizing clinical tumor samples. Moreover, our analysis revealed substantial CXCL12 expression differences between malignant and normal tissues, suggesting its potential correlation with prognosis. These findings indicate that CXCL12 is a distinct prognostic indicator in multiple tumors, necessitating further exploration due to divergent expression profiles across different tumor entities. Additionally, an unbalanced Th17/Treg cell ratio has been implicated in promoting inflammation and EAOC progression concurrently.

## Data Availability

The original contributions presented in the study are included in the article/supplementary material. Further inquiries can be directed to the corresponding author/s.
